# Asking the right questions for mutagenicity prediction from BioMedical text

**DOI:** 10.1038/s41540-023-00324-2

**Published:** 2023-12-18

**Authors:** Sathwik Acharya, Nicolas K. Shinada, Naoki Koyama, Megumi Ikemori, Tomoki Nishioka, Seiji Hitaoka, Atsushi Hakura, Shoji Asakura, Yukiko Matsuoka, Sucheendra K. Palaniappan

**Affiliations:** 1 The Systems Biology Institute, Tokyo, Japan; 2 SBX Corporation, Tokyo, Japan; 3 Global Drug Safety, Eisai Co., Ltd., Tokyo, Japan; 4 Planning Operation, hhc Data Creation Center, Eisai Co., Ltd., Tokyo, Japan; 5 5D Integration Unit, hhc Data Creation Center, Eisai Co., Ltd., Tokyo, Japan

**Keywords:** Computational biology and bioinformatics, Systems biology

## Abstract

Assessing the mutagenicity of chemicals is an essential task in the drug development process. Usually, databases and other structured sources for AMES mutagenicity exist, which have been carefully and laboriously curated from scientific publications. As knowledge accumulates over time, updating these databases is always an overhead and impractical. In this paper, we first propose the problem of predicting the mutagenicity of chemicals from textual information in scientific publications. More simply, given a chemical and evidence in the natural language form from publications where the mutagenicity of the chemical is described, the goal of the model/algorithm is to predict if it is potentially mutagenic or not. For this, we first construct a golden standard data set and then propose *MutaPredBERT*, a prediction model fine-tuned on *BioLinkBERT* based on a question-answering formulation of the problem. We leverage transfer learning and use the help of large transformer-based models to achieve a Macro F1 score of >0.88 even with relatively small data for fine-tuning. Our work establishes the utility of large language models for the construction of structured sources of knowledge bases directly from scientific publications.

## Introduction

Mutagenicity assessment of chemicals (drug products) is an important step in drug development process. Usually, the mutagenicity potential of chemical substances is assessed using in vitro gene mutation assays such as Ames test^1^ and the mouse lymphoma Tk mutation assay (MLA). Specifically, the Ames test is a bacterial assay that assesses the mutagenic potential of a chemical substance by using it on different strains of bacteria (*Salmonella typhimurium*) such as TA98, TA100, TA102, etc., and *Escherichiacoli* WP2 uvrA and WP2 uvrA/pKM101, and examining if they result in changes in the DNA of the organism (base-pair substitutions, frameshifts, insertions, and deletions). A positive test indicates that a chemical is mutagenic. Usually, regulatory guidelines such as those by OECD, and International Conference on Harmonization (ICH) guidelines are followed for genotoxicity testing methods that help evaluate the effect of chemical substances on human health. Also, ICH M7 guidelines for the assessment and control of mutagenic impurities in pharmaceuticals require mutagenicity tests to be an important prerequisite for regulatory approvals.

Owing to the ever-expanding repertoire of chemicals/virtual libraries and practical issues in keeping pace with their growth combined with the difficulty of isolating pure forms of chemicals (without impurities), regulatory authorities/researchers often turn to in silico methods such as Quantitative Structure-Activity Relationship (QSAR) ^2^ for mutagenicity assessment of chemicals. While these methods do not rely on conducting (the often time-consuming and expensive) experimental tests such as Ames, they rely on previous Ames mutagenicity results and chemical structure properties of the chemical for predicting the mutagenic potential of chemicals. Computational and machine learning models have played a key role in advancing this discipline^3,4^ by focusing on understanding the chemical, structural, and functional properties of chemicals that contribute to mutagenicity.

Most of the QSAR based tools often look up previously known mutagenicity results of chemicals which in turn are obtained from previously published scientific literature. Often, scientific publications in this field discuss the mutagenicity of chemical(s) in Ames assay in natural language. Consequently, an expert would comprehend the publications and compile them into data repositories that are used in the QSAR softwares. Keeping such information updated as more publications get added is a challenge in addition to manual effort in comprehending and making such structured data repositories. Machine learning models, especially from the disciple of natural language processing (NLP) can help in this regard. In fact NLP has seen several breakthroughs in the last few years and can be optimally adapted and leveraged for applications in bio-medicine^5,6^.

With this background, we first formulate the problem of predicting the mutagenicity of a chemical given the text that describes its mutagenicity as an NLP task. Given the lack of prior training data/ benchmarks, we first start by creating a golden standard data set for the task. After this, we create an end-to-end pipeline for training a model that can predict the mutagenicity of a chemical given the text which describes it. To do so, we have proposed a question-answering model based on transfer learning on large language models. These large language models are often based on transformers pretrained on large-scale natural text. We tried multiple language models before getting the best results on the model trained on the Stanford *BioLinkBERTbase* model^7^, a model pre-trained on large-scale scientific text from sources such as PubMed. Our final trained model, named *MutaPredBERT* can achieve an accuracy of >88 percent with a macro average F1 score of >0.88.

Our final goal with this study is for machine learning models to be able to continuously update the mutagenicity results from publications into high-quality data sources. We also hope that fellow researchers would leverage on the generated data set and our methodology to further improve the accuracy of this task. The big picture that springs from this task is that it paves the way for the construction of structured biomedical knowledge repositories from unstructured text that would ultimately help in the creation of an engine for scientific discovery. This engine is part of the endeavors of the Nobel Turing Challenge^8^ whose grand goal is to use such AI-based engines to generate new hypotheses in the biomedical sphere.

## Results

### Problem definition and formulation

**Fig. 1:**
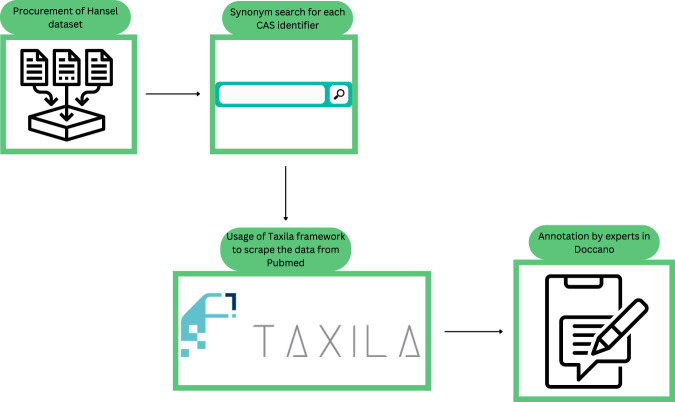
Overview of workflow used for abstract collection and annotation.

Our goal is to determine the mutagenic potential^9^ (mutagenicity) of a chemical entity from text which is described as its mutagenic potential. Typically, we could formulate this as a classification problem, where given a context text (passage) *C* = {*c*_1_, *c*_2_, . . , *c*_*e*_, . . , *c*_*m*_} and the chemical entity *c*_*e*_, we need to ascertain if *c*_*e*_ is mutagenic, *yes* or *no*. The problem is illustrated in Fig. 1.

**Fig. 2:**
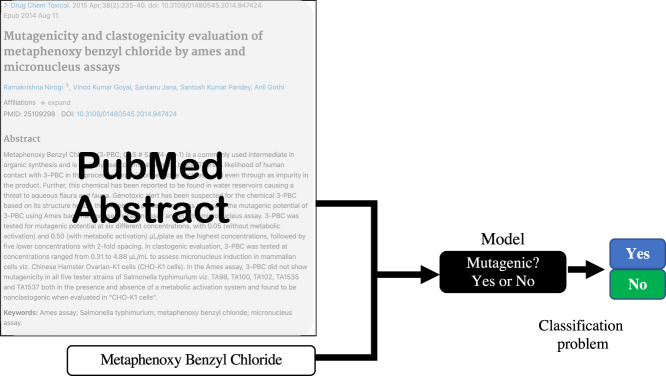
Graphical illustration of the problem formulation for mutagenicity classification.

While this problem can be tackled in multiple ways, we formulate it as a question-answering (QnA) task with a “*yes*", “*no*" outcome. For this, our setup consists of first synthesizing a question *Q*, for the context passage *C* such that the answer to the question *Q* will tell us if the compound *c*_*e*_ is mutagenic or not. The overview of this approach is shown in Fig. 2. Additionally, owing to the limited training data set available, we resort to transfer learning. We considered multiple domain-independent and domain-specific models as the base model which are then fine-tuned using the training data we collected for this task. Finally, our trained model *MutaPredBERT* performs well for the task.

### Performance

**Fig. 3:**
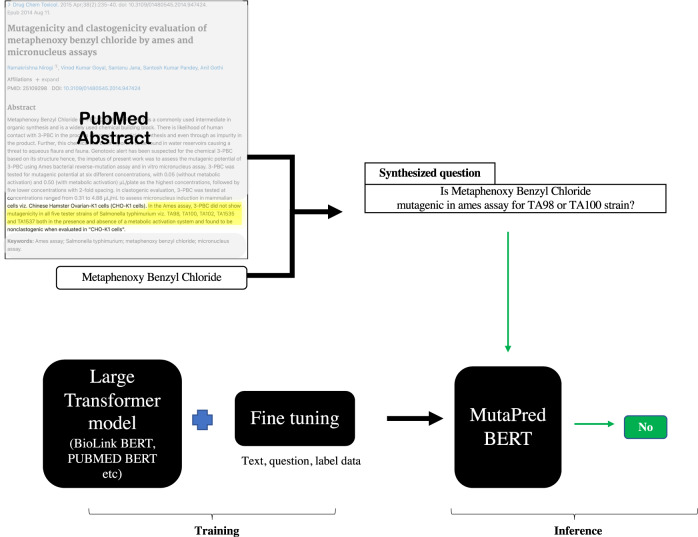
Graphical overview of our solution strategy and corresponding model flow.

Our proposed pipeline of a question-answering system for mutagenicity prediction can be divided into the following parts. First, we collect the training data, and formulate the QnA problem as described previously, followed by fine-tuning of a large language model and further analysis. The pipeline is schematically represented in Fig. 3.

As for the results, we start our experimental setup by first considering the performance of domain-specific models without any fine-tuning, i.e., using the model as is and checking the predictions. Our performance evaluations of the models were based on a 5 fold cross-validation strategy. The metrics used were the accuracy, the macro average F1 score and the weighted average F1 score.

As expected domain-specific models without any fine-tuning did not perform well as shown in Table 1. It showed that using large language models as is did not work and that fine-tuning was an essential step for improved accuracy.

**Table 1:** Performance metrics of different models and fine-tuning.

Model	Accuracy score	Macro Average F1 score	Weighted Average F1 score	domain-specific model
BioMegatron-uncased (no finetune)	50.238	0.494	0.5006	Yes
PubMedBERT (no finetune)	44.949	0.355	0.329	Yes
BioLinkBERT-base (no finetune)	55.367	0.357	0.395	Yes
Bert-large-uncased (fine-tuned)	75.882 ± 0.105	0.719 ± 0.182	0.727 ± 0.169	No
Deberta-large (fine-tuned)	55.650 ± 0.019	0.357 ± 0.008	0.398 ± 0.023	No
PubMedBERT (fine-tuned)	72.418 ± 0.0805	0.6982 ± 0.093	0.7038 ± 0.094	Yes
BioMegatron-uncased (fine-tuned)	86.028 ± 0.027	0.856 ± 0.026	0.859 ± 0.027	Yes
MutaPredBERT* BioLinkBERT-base (fine-tuned)	**88.513** ± **0.025**	**0.8815** ± **0.025**	**0.8837** ± **0.026**	Yes

Our model, MutaPredBERT, produced the best in class accuracy (as highlighted in bold in the above table).

Next, we wanted to check the efficiency of transfer learning based on fune-tuning using the golden standard data set for performance improvements. For this, we tried several large language models ranging from generic models (*BERT*^10^, *DeBERTa*^11^) to bio-medicine domain-specific models (BioMegatron^12^, *PubMedBERT*^13^, and BioLinkBERT-base). We followed the pipeline as described in the previous sections.

Domain-specific fine-tuned models show much better performance. For instance, BioMegatron and BioLinkBERT models which were pre-trained on PubMed abstracts and other biomedical related tasks show much better performance when fine-tuned on our task. Apart from the way in which these model were constructed, the other reason for the better performance lies in their ability to capture the domain information in their pretrained layers. This performance is in stark contrast with transformer-based models such as *DeBERTa* and *BERT* which were pre-trained on the general domain of SQUAD^14^ data set. Within the domain specific models, there are key aspects which could explain the variations of the performance of these models. *PubMedBERT* and BioLinkBERT are originally pre-trained on the PubMed corpus. *PubMedBERT* uses Masked Language Modeling (MLM), while BioLinkBERT employs MLM and Document Relation Prediction (DRP) to enhance pretraining, leading to improved performance. In contrast, BioMegatron, based on the Megatron-LM architecture, utilizes a large language model optimized for efficient distributed system training. Despite being pretrained on domain-specific data, BioMegatron uses a general vocabulary from Wikipedia and Books corpus, possibly explaining its slightly lower performance compared to MutaPredBERT. Please refer to Table 1 for details. Specifically, fine-tuned models based on BioMegatron and BioLinkBERT-base produced the best results, among them we used the model trained on *BioLinkBERT-base* as the base for our final model (*MutaPredBERT*).

**Fig. 4:**
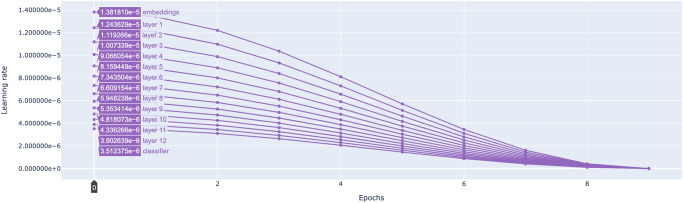
Plot showing the progression of the learning rate in each layer of the MutaPredBERT model with successive epochs. The initial learning rate set in the top layer is 1.3818e-05.

For training the *MutaPredBERT* model, to find the optimal learning rate hyper-parameter, we employed a hyper-parameter search using Optuna before being applied in the fine-tuning stage (please see methods section for more details). The hyper-parameter, being a continuous parameter was sampled from the range [1e−5, 5e−5] in the log domain. The process of hyper-parameter tuning was subject to 5 ‘trials’ or 5 different experiments and the optimal learning rate was 1.3818e−05 and the optimal number of epochs(the other hyper-parameter that was subject to optimization) was 9. Next, we started with the above-mentioned learning rate for the top layer and using LLRD, as described in the previous section, sequentially decrease it as we progress down each layer. Figure 4 shows the decay of the learning rate for each layer of the model.

### Explainability via per-layer embedding visualization

**Fig. 5:**
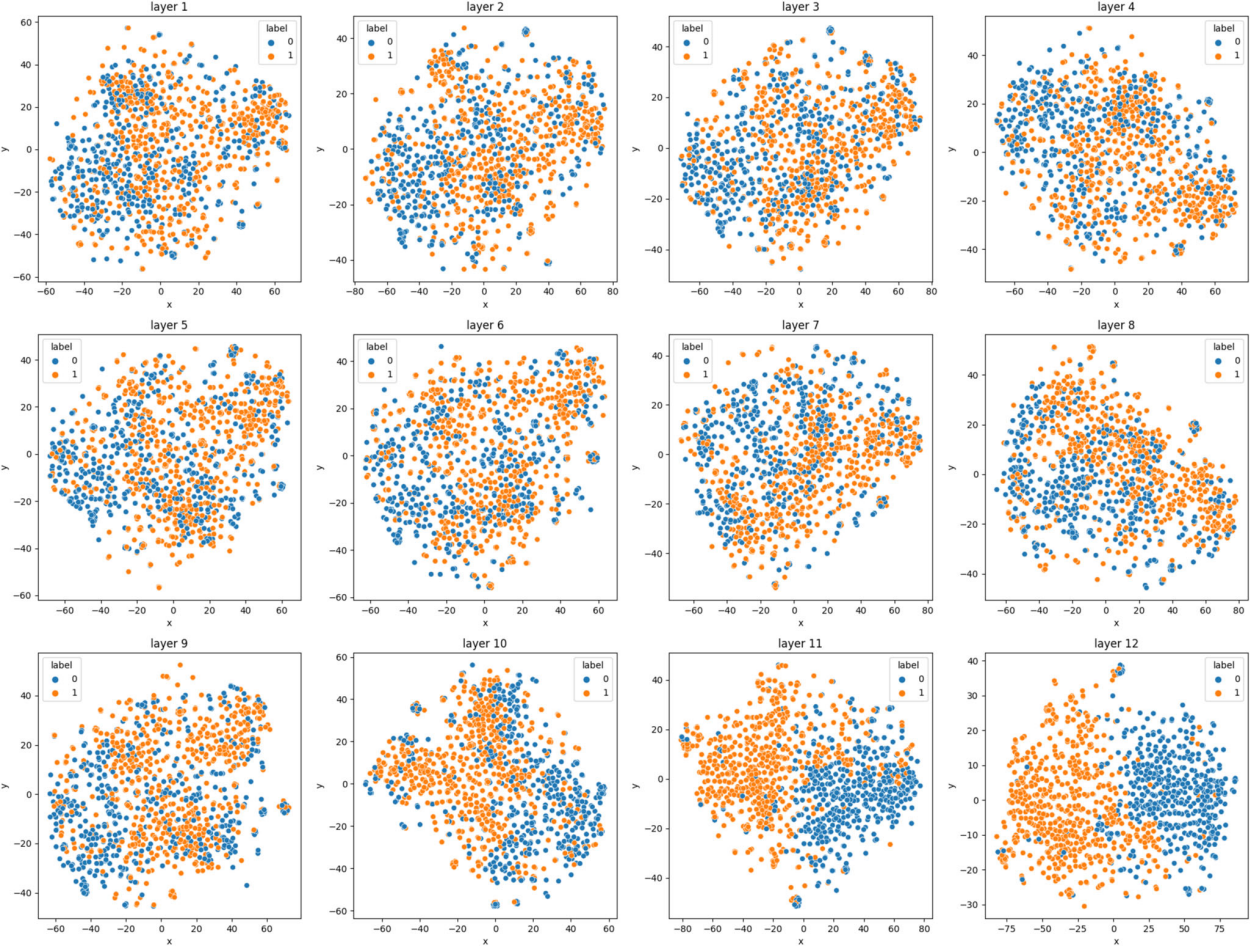
visualization of per-layer embedding space reduced to 2 dimensions.

To further validate the proposed methodology, we visualize the embedding space of each layer in the best performing *MutaPredBERT* model as training proceeds. The idea here is to see how well each of the stacked encoders in each layer successively builds upon the patterns highlighted by the previous layer and how it extracts different features from the input. As the default embedding dimension of the BioLinkBERT-base model is 768, it is necessary to use a feature reduction algorithm such as PCA^15^ or T-SNE^16^. We have used T-SNE here to reduce this dimension space to 2 dimensions and visualize the same. As can be seen in Fig. 5, with the progression of training, the model can capture well the semantics of the text and task at hand and gradually separate the embedding space into two possible clusters which indicate the two labels of the task at hand - exhibits mutagenic property or does not exhibit mutagenic property.

### Explainability via SHAP values (SHapley Additive exPlanations)

With the advent of transformer-based models, a well-known drawback of them is the black box nature of their working. To validate the question-answering approach we had proposed here for this task, it became essential to provide explainability of these models. To do this, we use SHAP values^17^ which were born out of cooperative game theory concepts where SHAP values are used to calculate the marginal feature importance over different coalitions.

When it comes to textual data (as it is here), the importance of each token is calculated by overlaying the original text that corresponds to that token. In our case when a chemical name and the corresponding abstract were passed to the model, the SHAP values indicate the portions of text that played a crucial role in the model’s predictions.

**Fig. 6:**
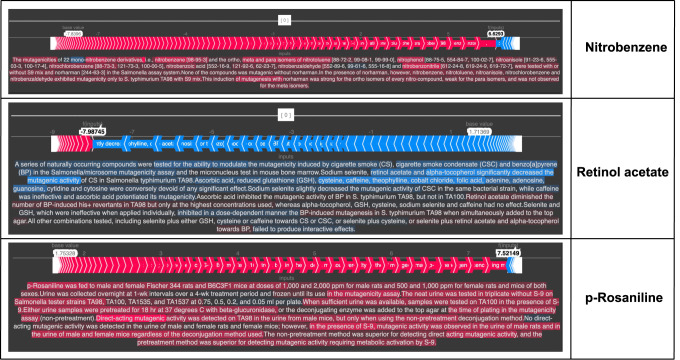
Demonstration of explainability via SHAP values. The chemicals of interest here are Nitrobenzene,p-Rosaniline, retinol acetate.

As for the explainability using SHAP values for *MutaPredBERT* model predictions (shown in Fig. 6), the abstract along with the chemical of interest is passed to the model with the QnA formulation. It must be noted that the regions of text highlighted in red indicate that it favors the prediction of mutagenic activity. Similarly, the regions of text highlighted in blue indicate that it favors the prediction of non-mutagenic activity. We picked three representative examples, Nitrobenzene, p-Rosaniline, and Retinol acetate to showcase. For p-Rosaniline, it can be seen that the correct context (such as *direct acting mutagenic activity*) was identified which helped in the model’s prediction. Similarly, for retinol acetate which non-mutagenic activity, we can note that the right phrases were identified. Another interesting case was nitrobenzene. This is an example where the MutaPredBERT model makes an incorrect prediction. Upon further examination, it can be seen that this chemical exhibits mutagenicity only in the presence of norharman. The model falters on this as the question that is prompted only asks whether nitrobenzene is mutagenic in the TA98 strain or not and does not mention norharman(which is unique to this abstract alone). Therefore, these SHAP values justify the proposed QnA methodology for mutagenicity prediction.

More importantly, the model files and the data set used in this paper have been made available on our supplementary website (more details in the supporting information section). Researchers and the community are invited to further improve the accuracy of models.

## Methods

This section details the different methodologies employed in the paper including preparation of training data,

### Training data preparation

Given that we did not have prior “gold-standard" training data for this task, we detail the steps of preparing the training data for this problem. In summary, we used a benchmark data set from Hansen et al. where each molecule structure is described using SMILES code and mutagenicity outcome defined as binary outcome (mutagens or non-mutagens)^18^. This data set was manually curated from a large collection of publications and safety agency reports and remains one of the most comprehensive public collections of empirical mutagenicity data collection to date. With each molecule’s structure described through its SMILES code in the dataset, we leveraged the Chemical Identifier Resolver (CIR) API from the NCI/CADD group to retrieve the different names, synonyms, and identifiers of the corresponding molecule. As such, for every molecule with Ames test results, we collect publication abstracts from which at least one of the molecule’s identifiers and mutagenicity-related keywords appears using the PubMed search APIs. After this, a manual review and re-annotation of all the collected abstracts is carried out to double-check the validity of the labels. In the end, the data set consists of publication abstracts, the chemical and bacterial species it describes, and their mutagenicity (labeled 0 for non-mutagens, and labeled 1 for mutagens). The following section details the steps.

### Data collection

#### Collection of relevant abstracts

We started by procuring relevant abstracts which describe chemical entities and their AMES mutagenicity. For this, we used the help of eutils services of PubMed^19^. The Ames mutagenicity benchmark data set for the chemicals and their mutagenicity comes from the publication by Hansen et.al., where the column “CAS identifier" were extracted and subsequently, a synonym search was performed for each molecule using the Chemical Identifier Resolver API provided by the NCI ^20^. For each synonym retrieved, we first query the PubMed database using the following query:

- *((Ames[TIAB] OR Mutagenicity[TIAB] OR Genotoxicity[TIAB]) AND (test[TIAB] OR assay[TIAB]))AND ((Bacteria OR “Salmonella typhimurium" OR “Escherichia coli") AND (TA100 OR TA98 OR TA1537 OR TA1535 OR TA102 OR WP2uvrA OR (WP2uvrA AND pKM101))) AND* (***synonym***)

to retrieve the corresponding abstract, we then assess that every element of the query is present in the AbstractText tag of the PubMed xml. The resulting abstract is matched with the mutagenicity outcome from the Hansen dataset. The backend module that facilitates this is the Taxila framework^21^. Taxila is an end-to-end analysis and intelligence platform which combines state-of-the-art natural language processing and natural language understanding (NLP/NLU) algorithms to analyze text in context. A total of 2,127 abstracts were retrieved using this step.

#### Expert review and re-annotation

Next, to ensure that the data set is of good quality, we manually reviewed each of the 2127 abstracts along with the assigned chemical name and its mutagenicity label. We used the annotation software Doccano^22^. We discarded abstracts that either do not refer to the query compound or do not give a proper indication of Ames test result. For mutagenic compounds, at least one sentence in the abstract should refer directly to the query compound’s mutagenicity. In the case of different outcomes that were tested in different conditions, we prioritize mutagenicity outcomes if any. For the non-mutagenic case, we looked for a clear textual relationship between the query compound and its lack of mutagenicity (or anti-mutagenicity property). Following this, our refined data set consisted of 1646 abstracts that were labeled with a 0 (730 abstracts) for non-mutagenic or a 1 (916 abstracts) for mutagenic, the remaining abstracts were discarded for further steps.

### Generating questions from abstracts

Given that we formulated the mutagenicity prediction as a question-answering task, it was important to annotate the obtained abstracts with the right questions to facilitate the question-answering model. With this in mind, a few samples were taken and manually annotated by experts with the right questions. A typical question can be seen in Fig. 2. The question usually follows the following format:

*Is (chemical name) mutagenic for (test name) in (strain names)?*

**Fig. 7:**
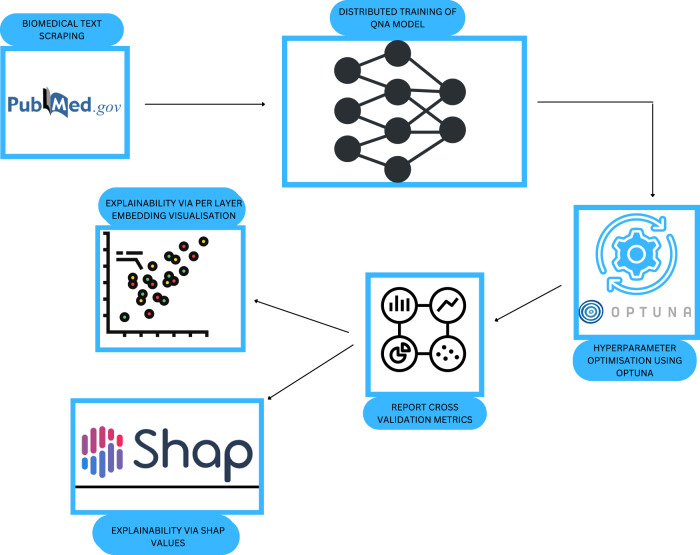
Overview of the pipeline used for the mutagenicity prediction task.

It is worth mentioning that we could use text generation models such as T5^23^ to generate the right questions given the abstract as context and speed up the annotation process. The workflow of the above two steps in the pipeline is illustrated in Fig. 7.

### Distributed training of the QnA model using the Accelerate framework

The crux of the entire training process relies on the ability to fine-tune large pre-trained language models for our task. Such large language models are usually based on transformer architecture and are usually trained on strategies such as masked learning on vast amounts of natural language data which opens up avenues for fine-tuning them for the task at hand. A Transformer works by performing a small, constant number of steps. In each step, it applies an attention mechanism^24^ to understand relationships between all words in a sentence, regardless of their respective position. In essence, it dynamically provides importance to a few key tokens in the input sequence by altering the token embeddings. Attention depends on three main concepts i.e the query weights (*Q*), the key weights (*K*) and the value weights (*V*) which leads to the attention formula as shown below:

1 $$\,{{\mbox{Attention}}}(Q,K,V)={{\mbox{softmax}}}\,\left(\frac{Q{K}^{T}}{\sqrt{{d}_{k}}}\right)V$$

Most *BERT* based models such as *DistilBERT*^25^, *ALBERT*^26^ build upon this to usher in the concept of Multi-Head Attention. A Multi-Head Attention Layer can be considered a stack of parallel Attention Layers. Each head in the Multi-Head Attention Layer intakes the new embedding (Positional Encoding generated in the last step). The output from all heads is then concatenated to produce a single output as shown below:

2 $$\,{{\mbox{MultiHead}}}(Q,K,V)={{\mbox{Concat}}}({{{\mbox{head}}}}_{1},\ldots ,{{{\mbox{head}}}}_{h}){W}^{O}$$

3 $$\,{{\mbox{where}}}\,\quad {{{\mbox{head}}}}_{i}={{\mbox{Attention}}}\,(Q{W}_{i}^{Q},K{W}_{i}^{K},V{W}_{i}^{V})$$

**Fig. 8:**
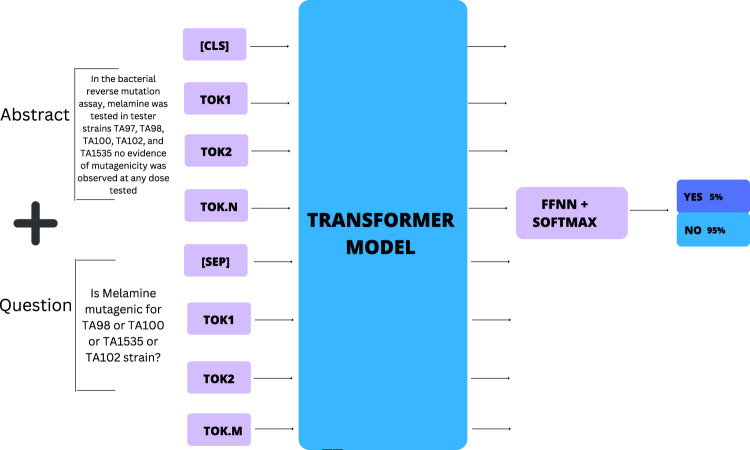
Demonstration of the Question-Answering approach used for fine-tuning purposes. The evidence (PubMed-abstract) and the corresponding question are passed as sequence pairs and separated by the [SEP] token. They are subsequently broken down into respective tokens by the tokenizer and fed to the transformer model wherein the prediction is made on the [CLS] or classifier token.

The optimizer used in this study was the Adam^27^ optimizer. This optimizer is usually the preferred one in deep learning applications due to its parameter initialization robustness, low memory requirements, efficiency, and convergence speed. In the typical fashion, the transformer-based models are loaded using the popular HuggingFace library^28^. Figure 8 shows the mechanism in which the abstract-question are taken in pairs for fine-tuning purposes. In our study, we have trained a variety of models including *BERT* (large), *DeBERTa*, *PubMedBERT*, BioLinkBERT. This phase has the typical tokenizing procedure done for the question-abstract pair which produces the token IDs and the attention mask. A maximum sequence length of 512 is maintained as this is what *BERT* based models are limited to. The truncation of the question-abstract pair is done to this maximum length. Since these models are very large (millions of parameters) and training would take longer in a single GPU setting, we have used a Distributed Data Parallel (DDP) mechanism using the Accelerate^29^ framework to divide the computational load between two GPUs thereby reducing the training time.

### Hyper-parameter tuning using Optuna

Hyper-parameter tuning is another essential task as we can boost the performance of our model with slight modifications in the training parameters. To do this with transformers models, we use the Optuna^30^ library to easily execute multiple runs in parallel and leverage different state-of-the-art tuning algorithms with minimal code changes. Although there are different strategies such as Grid Search^31^, and Bayesian optimization^32^, Optuna follows a technique called Tree-Parzen estimator^33^ which selects the hyper-parameters from the search space based on the history of trials conducted. This dynamic construction of search space coupled with efficient sampling and pruning algorithms and easy parallelization makes it ideal for this use case. The two main hyper-parameters that were tuned are the learning rate and the number of epochs. The objective function that was chosen to maximize here was the validation accuracy.

### Increasing few-sample fine-tuning stability via optimization techniques

Considering the low number of samples we have in our data set (1635 samples), we tried different optimization techniques to boost the performance. One such technique is the Layer-wise Learning Rate Decay (LLRD). LLRD^34^ is a method that applies higher learning rates for top layers and lower learning rates for bottom layers. This is accomplished by setting the learning rate of the top layer and using a multiplicative decay rate to decrease the learning rate layer-by-layer from top to bottom. The goal is to modify the lower layers that encode more general information less than the top layers that are more specific to the pre-training task. This technique of LLRD has been used in all the models that are bench marked during the fine-tuning stage.

## Discussion

In this work we have proposed the mutagenicity prediction problem from scientific publications (natural language). The problem comprises of predicting the mutagenicity of a chemical given the text that describes its mutagenicity. We have prepared a golden standard data set for this challenge. We also formulated the problem as a question-answering problem. Next, we leveraged pretrained large language models and a relatively small training data set to train a model, *MutaPredBERT* which achieves a very good accuracy for the task. The model was fine-tuned from the *BioLinkBERT* − *base* model using transfer learning. We utilize multiple optimization strategies for optimal learning outcomes in this work. We hope that this paper will further lead to research progress on bettering the accuracy of such models. In addition, for the field of toxicology, such models can be used to constantly update and build structured knowledge bases that can be used for crucial tasks in the drug development process.

In terms of future work, there are several possible improvements. For instance, for generating questions in our pipeline we used a template-filling approach in this work since it achieved good accuracy, however, we could leverage natural language generation models such as T5 to generate the right questions given the abstract as context and speed up the annotation process. Similarly, to increase the data set size for training, data augmentation strategies such as the paraphrasing method and the back translation method can be tried. For instance, pegasus^35^ T5 backbone can be used for paraphrasing whereas the back translation method relying on translating text data to another language and then translating it back to the original language can be tried. This technique allows generating of textual data of distinct wording to the original text while preserving the original context and meaning.

One could also look into the avenues of using an ensemble of models for better accuracy scores in the inference stage. Techniques such as Stochastic Weighting Average (SWA)^36^, and mix-out regularization^37^ also seem promising to combat the problem of over-fitting, especially when dealing with smaller data sets for learning tasks. In addition, one crucial area of improvement is to look at full text of publications, rather than just abstracts of publications.

### Reporting summary

Further information on research design is available in the Nature Research Reporting Summary linked to this article.

### Supplementary information

Reporting summary

## Data Availability

All the data for training models, the training data used in the paper are available in the supplementary website here.

## Code availability

All the code for training models, the training data used in the paper are available in the supplementary website here.
